# Investigating peripheral blood monocyte and T-cell subsets as non-invasive biomarkers for asymptomatic hepatic steatosis: results from the Multi-Ethnic Study of Atherosclerosis

**DOI:** 10.3389/fimmu.2024.1243526

**Published:** 2024-03-26

**Authors:** Rhys W. Niedecker, Joseph A. Delaney, Margaret F. Doyle, Andrew D. Sparks, Colleen M. Sitlani, Petra Buzkova, Irfan Zeb, Russell P. Tracy, Bruce M. Psaty, Matthew J. Budoff, Nels C. Olson

**Affiliations:** ^1^ Department of Pathology and Laboratory Medicine, Larner College of Medicine, University of Vermont, Burlington, VT, United States; ^2^ General Internal Medicine, University of Washington, Seattle, WA, United States; ^3^ Department of Medical Biostatistics, Larner College of Medicine, University of Vermont, Burlington, VT, United States; ^4^ Cardiovascular Health Research Unit, Department of Medicine, University of Washington, Seattle, WA, United States; ^5^ Department of Biostatistics, University of Washington School of Public Health, Seattle, WA, United States; ^6^ Department of Medicine, West Virginia University Heart and Vascular Institute, Morgantown, WV, United States; ^7^ Department of Biochemistry, Larner College of Medicine, University of Vermont, Burlington, VT, United States; ^8^ Department of Epidemiology, University of Washington, Seattle, WA, United States; ^9^ Department of Health Systems and Population Health, University of Washington, Seattle, WA, United States; ^10^ Lundquist Institute at Harbor-UCLA Medical Center, Torrance, CA, United States

**Keywords:** hepatic attenuation, liver-spleen index, metabolic dysfunction-associated fatty liver disease, computed tomography, T cells, peripheral blood monocular cells

## Abstract

**Background:**

Circulating immune cells have gained interest as biomarkers of hepatic steatosis. Data on the relationships between immune cell subsets and early-stage steatosis in population-based cohorts are limited.

**Methods:**

This study included 1,944 asymptomatic participants of the Multi-Ethnic Study of Atherosclerosis (MESA) with immune cell phenotyping and computed tomography measures of liver fat. Participants with heavy alcohol use were excluded. A liver-to-spleen ratio Hounsfield units (HU) <1.0 and liver attenuation <40 HU were used to diagnose liver fat presence and >30% liver fat content, respectively. Logistic regression estimated cross-sectional associations of immune cell subsets with liver fat parameters adjusted for risk factors. We hypothesized that higher proportions of non-classical monocytes, Th1, Th17, and memory CD4^+^ T cells, and lower proportions of classical monocytes and naive CD4^+^ T cells, were associated with liver fat. Exploratory analyses evaluated additional immune cell phenotypes (n = 19).

**Results:**

None of the hypothesized cells were associated with presence of liver fat. Higher memory CD4^+^ T cells were associated with >30% liver fat content, but this was not significant after correction for multiple hypothesis testing (odds ratio (OR): 1.31, 95% confidence interval (CI): 1.03, 1.66). In exploratory analyses unadjusted for multiple testing, higher proportions of CD8^+^CD57^+^ T cells were associated with liver fat presence (OR: 1.21, 95% CI: 1.02, 1.44) and >30% liver fat content (OR: 1.34, 95% CI: 1.07, 1.69).

**Conclusions:**

Higher circulating memory CD4^+^ T cells may reflect liver fat severity. CD8^+^CD57^+^ cells were associated with liver fat presence and severity, but replication of findings is required.

## 1 Introduction

Metabolic dysfunction-associated fatty liver disease (MAFLD) is a common chronic hepatic disease, affecting an estimated 30% of adults in the United States and 37% of the global population (1, 2). MAFLD encompasses a spectrum of disease states from hepatic steatosis to metabolic dysfunction-associated steatohepatitis (MASH) and progression to fibrosis, cirrhosis, and hepatocellular carcinoma (3). An estimated 20% of patients with MAFLD progress to MASH, with approximately 20% of MASH patients progressing to advanced fibrosis (4).

Several cardiometabolic conditions characterized by chronic inflammation, such as obesity, dyslipidemia, insulin resistance, and type 2 diabetes commonly coexist with MAFLD. In turn, MAFLD increases the risk of many chronic inflammatory conditions including insulin resistance, type 2 diabetes, atherosclerosis, and cardiovascular diseases (CVD) (5–8). Lipotoxicity, oxidative stress, and proinflammatory mediator release are important features driving progression of hepatic steatosis to MASH (5, 9, 10).

Liver inflammation during MAFLD is initiated by hepatocytes, liver sinusoidal endothelial cells, and Kupffer cells in response to metabolic dysfunction (5). Activation of these cells during steatosis results in cytokine and chemokine secretion, furthering tissue injury and promoting recruitment of monocytes, lymphocytes, and T cells to the liver. Immune cell infiltration exacerbates proinflammatory mediator release, creating a feedback mechanism that promotes further immune cell recruitment and drives lobular inflammation characteristic of MASH.

Several lines of experimental evidence suggest involvement of innate and adaptive immune cells in MAFLD pathogenesis and the progression from hepatic steatosis to MASH (5, 9, 10). Monocytes and CD4^+^ and CD8^+^ T cells are recruited to the liver during steatohepatitis onset, and mice deficient in these cells are protected against steatosis, parenchymal injury, and lobular inflammation induced by a high-fat diet (11–14). Similarly, mice deficient in IFN-γ and IL-17A are protected against liver injury and hepatic fibrosis in mouse models of MASH (15–17). These studies demonstrate roles of immune cells in experimental MAFLD models and suggest their involvement in MAFLD progression in humans.

Several studies have reported differences in the frequencies and functions of innate and adaptive immune cells in liver biopsy and peripheral blood samples of patients with MAFLD compared with those without disease (18–23). Studies of patients with severe fatty liver disease, however, may not be generalizable to asymptomatic individuals with subclinical disease. There is currently a paucity of data on the relationships between circulating immune cells and hepatic steatosis from population-based cohorts. Investigation of immune cell profiles in observational, community-based cohorts may aid in the identification of biomarkers associated with the early stages of MAFLD. In the present study, we evaluated the associations of immune cell subsets in peripheral blood with measures of liver fat assessed by computed tomography (CT) scans in asymptomatic participants of the community-based, Multi-Ethnic Study of Atherosclerosis (MESA).

## 2 Methods

### 2.1 Cohort

MESA is an epidemiological cohort study of 6,814 participants recruited in 2000–2002, aged 45–84 years (24). Participants were enrolled from six U.S. communities (Baltimore, MD; Chicago, IL; Forsyth County, NC; Los Angeles, CA; Manhattan, NY; and St. Paul, MN) and self-identified as either White, Black, Hispanic, or Chinese American. Those with a self-reported medical history of CVD, undergoing active treatments for cancer, pregnancy, or amputation met exclusion criteria for the study. MESA’s study design and procedure details are published (24). Participants answered standardized questionnaires, underwent assessment for CVD risk factors, completed CT imaging, and provided biospecimens.

The current study leverages data from a nested case–cohort study designed to evaluate relationships of circulating immune cell proportions with incident coronary heart disease (CHD) and heart failure (HF) events (25, 26). The case–cohort study sampled all participants with incident CHD and HF and a random cohort (n = 2,200). We leveraged this data for a secondary cross-sectional analysis of liver fat.

All procedures were conducted under institutionally approved protocols for human subjects research, and all participants provided written informed consent for study participation.

### 2.2 Cellular phenotyping

During the MESA baseline examination (2000–2002), peripheral blood mononuclear cells (PBMCs) were isolated from 8-mL citrate CPT tubes according to the manufacturer’s instructions (BD Biosciences) and cryopreserved at −135°C in media containing 90% fetal bovine serum and 10% dimethyl sulfoxide. Detailed methods, reagents, and flow cytometry gating strategies used for immune cell phenotyping are published (25, 27).

All antibodies were from Miltenyi Biotec and diluted according to the manufacturer’s recommendations. Cells were thawed quickly at 37°C and immediately diluted by the slow addition of media. Cell surface labeling was used to phenotype monocyte subsets, natural killer cells, γδ T cells, B cell subsets, and CD4^+^ and CD8^+^ naive, memory, differentiated, and CD45RA^+^ reexpressing effector memory cells (TEMRA). Surface labeling was performed by incubating cells with antibodies in the dark for 15 min at room temperature. Cells were centrifuged and washed with buffer, and the final pellet was placed in 1% paraformaldehyde and stored at 4°C until flow cytometry analysis. Monocytes were defined by surface labeling for CD14. Classical monocytes were defined as CD14^++^CD16^-^ and non-classical monocytes as CD14^+^CD16^++^. T helper cells and cytotoxic T cells were identified by surface labeling for CD4 and CD8, respectively. Naive and memory CD4^+^/CD8^+^ T cells were defined by cell surface staining of CD45RA^+^ and CD45RO^+^, respectively.

Phenotyping for Th1 (defined as CD4^+^IFN-γ^+^), Th2 (CD4^+^IL-4^+^), Th17 (CD4^+^IL-17A^+^), T cytotoxic type 1 (Tc1; defined as CD8^+^IFN-γ^+^), Tc2 (CD8^+^IL-4^+^), and Tc17 (CD8^+^IL-17A^+^) cells was performed using intracellular cytokine staining as described in detail previously (27). Cells were activated for 3 h at 37°C with phorbol myristate acetate and ionomycin in the presence of Brefeldin A to inhibit cytokine secretion. Cells were subsequently incubated with anti-CD4 and anti-CD8 antibodies for 15 min in the dark at room temperature. Following a wash and fixation with 2% paraformaldehyde, cells were permeabilized using 0.1% saponin and incubated in the dark with anti-IFN-γ, anti-IL-4, and anti-IL-17A antibodies for 15 min at room temperature. Cells were washed, fixed in 2% paraformaldehyde, and stored at 4°C in the dark until analysis.

Cell phenotyping was performed by flow cytometry analysis using a MACSQuant 10 and MACSQuantify software (Miltenyi Biotec). An average of 41,800 PBMCs were evaluated per participant. Single-color controls were used for compensation and isotype controls for setting negative gates (25). Cell phenotypes were expressed as proportions of their parent populations (e.g., monocytes as a percentage of CD14^+^ cells, T helper cells as a percentage of CD4^+^, and cytotoxic T cells as a percentage of CD8^+^ cells). All cell phenotypes included in the study, the molecular markers used to define them, how they were expressed, and their means and standard deviations are presented in **Supplementary Table 1** in the **Supplementary Material**.

### 2.3 Laboratory measurements and definitions

Demographic and alcohol use information was obtained by standardized questionnaire at the baseline study exam (24). Education was categorized as less than college degree or college degree or above. Serum glucose was measured by the Vitros analyzer (Johnson & Johnson Clinical Diagnostics, Inc.). Diabetes was defined as a fasting blood glucose ≥126 mg/dL or use of insulin or oral hypoglycemic medication. Waist circumference was measured at the level of the umbilicus in cm. Lipid measurements were performed as described (24). Cytomegalovirus (CMV) IgG antibodies were measured in serum samples by enzyme immunoassay (Diamedix Corp.) (coefficients of variation 5.1%–6.8%).

### 2.4 Liver fat measurements

Non-enhanced cardiac CT scans were performed at the MESA baseline examination in all participants (28). CT scans were used to assess for liver fat as described in detail (29).

Hepatic and splenic Hounsfield unit (HU) attenuation values were measured using two regions of interest (ROI) greater than 100 mm^2^ in the right liver lobe anterioposteriorly, one in the left liver lobe and one ROI in the spleen. Attenuation measurements of both right and left liver lobes were available in 6,464 scans. Spleen attenuation measurements were available in 4,396 scans. Liver and spleen HU attenuation measurements were highly reproducible with intra- and inter-reader correlations of 0.96–0.99 (29). A liver/spleen attenuation (L/S) ratio was calculated by dividing the mean HU measurements of both right liver lobe ROIs by the spleen HU measurement. An L/S ratio <1.0 was used as the diagnosis of the presence of liver fat (29). A liver attenuation <40 HU was assessed as a cutoff of >30% liver fat content (14, 30).

### 2.5 Statistical analysis

All data used in the analyses were from the MESA baseline exam (2000–2002). Analyses included all participants with at least one immune cell phenotype and liver attenuation data (n = 2,133). Participants with heavy alcohol use (n = 189), defined as >14 drinks per week for men and >7 drinks per week for women, were excluded from the study. Following exclusions, 1,944 participants were included in the analysis; liver attenuation data were available in 1,944 participants, and L/S ratio data were available in 1,316 participants. **Supplementary Table 1** presents the number of participants with data for each of the immune cell assays. The number of cellular phenotypes measured per participant varied due to occasional poor sample quality or technical errors.

Informed by the literature (10, 12, 15, 18–21) and prior results in MESA (31–33), we specified *a priori* hypotheses for six immune cell subsets. We hypothesized that higher proportions of non-classical monocytes (CD14^+^CD16^++^), Th1 (CD4^+^IFN-γ^+^), Th17 (CD4^+^IL-17A^+^), and memory CD4^+^ (CD4^+^CD45RO^+^) cells, and lower proportions of classical monocytes (CD14^++^CD16^−^) and naive CD4^+^ cells (CD4^+^CD45RA^+^), would be associated with a higher prevalence of liver fat. As exploratory analyses, we assessed relationships of 19 additional immune cell subsets phenotyped in the parent case–cohort study (25). The primary study outcome was an L/S ratio <1/0. Liver attenuation <40 HU was assessed as a secondary outcome.

Logistic regression models were used to estimate cross-sectional associations of immune cell subsets with liver fat parameters. Immune cells were analyzed in separate models per 1-standard deviation (SD) increment. Models included case–cohort weights that accounted for the study design which oversampled CHD and HF cases. Models were adjusted for demographic variables (age, sex, race/ethnicity, education, and cell phenotyping analytical batch) and subsequently for waist circumference, alcohol use, diabetes status, systolic blood pressure, hypertensive medication use, HDL, LDL, statin use, and triglycerides. Sensitivity analyses included additional adjustment for CMV antibody titers due to the known influence of CMV on T-cell immunity (34, 35). For the six *a priori* hypotheses, a Bonferroni correction was used to account for multiple hypothesis testing; statistical significance was defined as p < 0.008 (reflecting a Bonferroni correction of 0.05/6). In exploratory analyses of additional cell subsets, we used p <0.05 as the significance threshold.

## 3 Results

Descriptive characteristics of the study population are presented in **Table 1**. Presence of liver fat, defined by an L/S ratio <1.0, was present among 18.5% (n = 243) of the participants. Liver attenuation <40 HU was observed in 130 participants (6.7%).

**Table 1:** Descriptive characteristics of the study population stratified by presence of liver fat as defined by a liver-to-spleen (L/S) ratio <1.0.

	L/S ratio >1.0 (n=1073)	L/S ratio <1.0 (n=243)
Age, years (mean, SD)	64.4 (10.5)	62.6 (10.0)
Sex (*n*, %)		
Male	491 (45.8%)	115 (47.3%)
Female	582 (54.2%)	128 (52.7%)
Race/Ethnicity (*n*, %)		
White	401 (37.4%)	81 (33.3%)
Black	350 (32.6%)	52 (21.4%)
Hispanic	202 (18.8%)	74 (30.5%)
Chinese	120 (11.2%)	36 (14.8%)
College education or above (*n*, %)	372 (34.8%)	78 (32.2%)
BMI, kg/m^2^ (mean, SD)	28.0 (5.3)	30.5 (5.2)
Waist circumference, cm (mean, SD)	97.9 (14.1)	104.4 (13.2)
Systolic blood pressure, mm Hg (mean, SD)	130 (23)	130 (22)
Hypertension medication use (n, %)	449 (41.9%)	115 (47.3%)
Total Cholesterol, mg/dL (mean, SD)	193 (34)	190 (37)
LDL cholesterol, mg/dL (mean, SD)	117 (30)	111 (29)
HDL cholesterol, mg/dL (mean, SD)	51 (15)	45 (12)
Triglycerides, mg/dL (median, IQR)	105 (76, 153)	155 (110, 218)
Statin use (*n*, %)	183 (17.1%)	41 (16.9%)
C-reactive protein, mg/L (median, IQR)	1.9 (0.9, 4.2)	2.5 (1.1, 6.1)
Cytomegalovirus, EU/mL (median, IQR)	117 (41, 225)	122 (54, 258)
Fasting glucose, mg/dL (median, IQR)	90 (83, 99)	98 (87, 113)
Diabetes (*n*, %)	153 (14.3%)	57 (23.5%)

BMI, body mass index; IQR, inter-quartile range; SD, standard deviation.

### 3.1 Associations of monocyte and T-cell subsets specified as *a priori* hypotheses with a liver-to-spleen ratio <1.0 and liver attenuation <40 HU

**Table 2** presents associations of the six cell subsets specified as *a priori* hypotheses (classical monocytes, non-classical monocytes, Th1, Th17, and memory and naive CD4^+^ T cells) with the presence of liver fat (assessed by an L/S ratio <1.0). None of these six subsets were associated with liver fat presence in either demographic or risk factor adjusted models (p < 0.05). In analyses of the secondary outcome, higher memory CD4^+^ cells (CD4^+^CD45RO^+^) were associated with a higher odds of liver attenuation <40 HU (indicating >30% liver fat content) in both models (odds ratio (OR): 1.31, 95% confidence interval (CI): 1.03, 1.66; p = 0.03 in risk factor-adjusted models), but this association did not meet the significance threshold for testing multiple comparisons (p < 0.008).

**Table 2:** Cross-sectional associations of immune cell subsets specified as primary hypotheses with liver-to-spleen ratio <1.0 and liver attenuation <40 HU.

Cell subset (1-SD value)	Liver-to-Spleen Ratio <1.0 (Model 1) OR (95% CI)	P value	Liver-to-Spleen Ratio <1.0 (Model 2) OR (95% CI)	P value	Liver Attenuation <40 HU (Model 1) OR (95% CI)	P value	Liver Attenuation <40 HU (Model 2) OR (95% CI)	P value
Classical Monocytes (CD14^++^CD16^-^) (10.4%)	0.86 (0.72, 1.02)	0.08	0.85 (0.69, 1.04)	0.11	0.97 (0.78, 1.23)	0.86	0.98 (0.75, 1.28)	0.87
Non-Classical Monocytes (CD14^+^CD16^++^) (7.8%)	1.14 (0.97, 1.33)	0.11	1.11 (0.92, 1.33)	0.27	1.02 (0.83, 1.28)	0.79	1.03 (0.79, 1.33)	0.84
Naive CD4^+^ (CD4^+^CD45RA^+^) (13.0%)	0.96 (0.81, 1.15)	0.69	0.99 (0.84, 1.19)	0.98	0.84 (0.68, 1.04)	0.11	0.81 (0.65, 1.01)	0.07
Memory CD4^+^ (CD4^+^CD45RO^+^) (14.9%)	1.11 (0.92, 1.35)	0.27	1.06 (0.88, 1.29)	0.53	1.28 (1.02, 1.60)	0.03	1.31 (1.03, 1.66)	0.03
Th1 (CD4^+^IFN-γ^+^) (7.9%)	1.04 (0.87, 1.24)	0.66	0.98 (0.81, 1.18)	0.80	0.97 (0.78, 1.20)	0.76	0.92 (0.73, 1.17)	0.52
Th17 (CD4^+^IL17-A^+^) (1.4%)	0.90 (0.73, 1.10)	0.30	0.88 (0.72, 1.07)	0.20	0.95 (0.76, 1.18)	0.62	1.01 (0.81, 1.25)	0.95

Analyses are by logistic regression models and include sampling weights to account for the case-cohort study design. Cells were analyzed in separate models per 1-SD higher value (shown in parentheses). An L/S ratio <1.0 was used as the diagnosis of the presence of liver fat. A liver attenuation <40 HU was assessed as a cutoff of >30% liver fat content.

Model 1: Age, sex, race/ethnicity, education, and analytical batch.

Model 2: Age, sex, race/ethnicity, education, analytical batch, waist circumference, alcohol use, diabetes status, statin use, hypertensive medication use, systolic blood pressure, HDL, LDL, and triglycerides.

### 3.2 Exploratory analyses of additional immune cell subsets with hepatic attenuation measurements

**Table 3** presents exploratory analyses assessing relationships of 19 additional immune cell subsets assayed in the parent case–cohort study with liver fat presence and >30% liver fat content. Adjusted for risk factors, a 1-SD higher proportion of CD8^+^CD57^+^ T cells was associated with a higher odds of liver fat presence (OR: 1.21, 95% CI: 1.02, 1.44) and >30% liver fat content (OR: 1.34, 95% CI: 1.07, 1.69), as defined using a significance threshold of p < 0.05. The association of CD8^+^CD57^+^ cells with >30% liver fat content was supported by relationships of the highly correlated phenotypes CD8^+^CD28^-^ (OR: 1.35, 95% CI: 1.05, 1.74) and CD8^+^CD28^-^CD57^+^ cells (OR: 1.37, 95% CI: 1.07, 1.76) with liver attenuation <40 HU (**Table 3**).

**Table 3:** Odds ratios of immune cell subsets included in exploratory analyses with liver-to-spleen ratio <1.0 and liver attenuation <40 HU.

Cell subset (1-SD value)	Liver-to-Spleen Ratio <1.0 (Model 1) OR (95% CI)	P value	Liver-to-Spleen Ratio <1.0 (Model 2) OR (95% CI)	P value	Liver Attenuation <40 HU (Model 1) OR (95% CI)	P value	Liver Attenuation <40 HU (Model 2) OR (95% CI)	P value
γδ (5.6%)	0.85 (0.71, 1.01)	0.06	0.84 (0.69, 1.01)	0.06	1.09 (0.89, 1.32)	0.41	1.16 (0.93, 1.45)	0.18
Natural Killer (5.8%)	0.95 (0.79, 1.14)	0.58	0.92 (0.74, 1.13)	0.42	0.95 (0.76, 1.18)	0.63	0.95 (0.74, 1.21)	0.67
Intermediate monocyte (CD14^+^CD16^+^) (7.4%)	1.09 (0.89, 1.35)	0.41	1.15 (0.90, 1.45)	0.26	0.99 (0.75, 1.33)	0.99	1.01 (0.73, 1.37)	0.98
Th2 (CD4^+^IL-4^+^) (1.8%)	0.92 (0.76, 1.12)	0.42	0.88 (0.71, 1.09)	0.27	1.12 (0.92, 1.37)	0.26	1.09 (0.88, 1.35)	0.44
Treg (CD4^+^CD25^+^CD127^-^) (2.2%)	0.81 (0.65, 1.01)	0.06	0.83 (0.65, 1.05)	0.12	1.13 (0.88, 1.44)	0.34	1.16 (0.87, 1.55)	0.31
CD4^+^CD25^+^ (12.6%)	1.09 (0.90, 1.32)	0.39	1.05 (0.85, 1.28)	0.67	1.13 (0.87, 1.46)	0.35	1.06 (0.81, 1.40)	0.67
CD4^+^CD28^-^ (9.8%)	0.87 (0.72, 1.06)	0.17	0.83 (0.67, 1.04)	0.10	0.99 (0.79, 1.23)	0.90	1.00 (0.77, 1.30)	0.99
CD4^+^CD57^+^ (11.3%)	0.97 (0.80, 1.17)	0.76	0.89 (0.73, 1.08)	0.24	1.06 (0.83, 1.35)	0.65	0.97 (0.74, 1.26)	0.80
CD4^+^CD28^-^CD57^+^ (8.1%)	0.93 (0.78, 1.10)	0.40	0.87 (0.71, 1.06)	0.17	0.99 (0.80, 1.22)	0.91	0.98 (0.87, 1.25)	0.87
CD4^+^ TEMRA (4.9%)	0.96 (0.80, 1.15)	0.66	0.86 (0.68, 1.09)	0.20	1.08 (0.86, 1.35)	0.50	1.01 (0.77, 1.32)	0.94
Tc1 (CD8^+^IFN-γ^+^) (17.7%)	0.98 (0.82, 1.17)	0.85	0.98 (0.79, 1.21)	0.87	0.90 (0.69, 1.17)	0.43	0.93 (0.68, 1.27)	0.66
Tc2 (CD8^+^IL-4^+^) (4.8%)	0.86 (0.69, 1.08)	0.18	0.83 (0.66, 1.05)	0.12	0.97 (0.74, 1.28)	0.85	1.00 (0.73, 1.39)	0.98
Tc17 (CD8^+^IL-17A^+^) (1.6%)	0.95 (0.79, 1.15)	0.59	0.93 (0.76, 1.14)	0.49	0.96 (0.73, 1.26)	0.77	1.00 (0.76, 1.31)	0.98
Naive CD8^+^ (CD8^+^CD45RA^+^) (15.6%)	1.01 (0.86, 1.19)	0.88	1.00 (0.84, 1.20)	0.96	1.08 (0.87, 1.34)	0.50	1.04 (0.81, 1.32)	0.77
Memory CD8^+^ (CD8^+^CD45RO^+^) (11.9%)	0.95 (0.81, 1.12)	0.57	0.94 (0.79, 1.11)	0.46	0.96 (0.78, 1.19)	0.71	0.98 (0.77, 1.25)	0.90
CD8^+^CD28^-^ (16.0%)	1.00 (0.86, 1.17)	1.00	1.04 (0.88, 1.24)	0.62	1.19 (0.95, 1.50)	0.13	1.35 (1.05, 1.74)	0.02
CD8^+^CD57^+^ (16.6%)	1.21 (1.03, 1.41)	0.02	1.21 (1.02, 1.44)	0.03	1.25 (1.02, 1.54)	0.03	1.34 (1.07, 1.69)	0.01
CD8^+^CD28^-^CD57^+^ (16.1%)	1.11 (0.96, 1.31)	0.15	1.14 (0.96, 1.36)	0.14	1.25 (1.00, 1.56)	0.05	1.37 (1.07, 1.76)	0.01
CD8^+^ TEMRA (14.6%)	1.08 (0.92, 1.26)	0.36	1.07 (0.89, 1.28)	0.48	1.19 (0.95, 1.48)	0.12	1.20 (0.94, 1.52)	0.14

Analyses are by logistic regression models and include sampling weights. Cells were analyzed in separate models per 1-SD higher value (shown in parentheses). An L/S ratio <1.0 was used as the diagnosis of the presence of liver fat. A liver attenuation <40 HU was assessed as a cutoff of >30% liver fat content. TEMRA, T effector memory RA^+^.

Model 1: Age, sex, race/ethnicity, education, and analytical batch.

Model 2: Age, sex, race/ethnicity, education, analytical batch, waist circumference, alcohol use, diabetes status, statin use, hypertensive medication use, systolic blood pressure, HDL, LDL, and triglycerides.

### 3.3 Sensitivity analyses adjusting for cytomegalovirus antibody titers

In sensitivity analyses that added CMV antibody titers as a covariate in risk factor models, the association of memory CD4^+^ cells with >30% liver fat content was slightly attenuated (OR: 1.27, 95% CI: 1.00, 1.61; p = 0.05). The relationship of CD8^+^CD57^+^ cells with liver fat presence was essentially the same following CMV adjustment, whereas the association with >30% liver fat content was accentuated (OR: 1.40, 95% CI: 1.11, 1.77; p = 0.005). The associations of CD8^+^CD28^−^ and CD8^+^CD28^-^CD57^+^ cells with >30% liver fat content were also accentuated following CMV adjustment (CD8^+^CD28^−^ OR: 1.45, 95% CI: 1.11, 1.90), p = 0.006; CD8^+^CD28^-^CD57^+^ OR: 1.47, 95% CI: 1.14, 1.90; p = 0.003). Interpretation of results was unchanged for all other cell phenotypes (presented in **Supplementary Table 2**). These results suggest the relationships of CD57 expressing CD8^+^ cells with hepatic steatosis were not driven by CMV infection.

## 4 Discussion

In this community-based study of asymptomatic adults, peripheral blood monocyte and CD4^+^ T helper cell subsets were not associated with the presence of liver fat as assessed using a liver/spleen attenuation ratio <1.0 by CT imaging. As hypothesized, higher memory CD4^+^ T cells were associated with a liver fat content >30%, but the association did not withstand adjustment for multiple hypothesis testing. In exploratory analyses, CD8^+^CD57^+^ T cells were associated with both the presence of liver fat and >30% liver fat content when not accounting for multiple comparisons.

Accumulating evidence has implicated roles of monocytes and lymphocytes in hepatic steatosis and the progression to MASH (9, 10, 36, 37). In animal models of diet-induced fatty liver disease, monocytes, Th1, and Th17 cells are recruited to the liver (11, 12, 38–40). Pharmacological inhibition of monocyte recruitment and genetic ablation of signature Th1- and Th17-associated cytokines protect against steatohepatitis (11, 15–17, 40). Increased intrahepatic and peripheral blood Th1 and Th17 cells have also been reported in patients with MASH (18, 19). Higher circulating levels of non-classical monocytes, and lower classical monocytes, were reported in MAFLD patients compared with controls (20, 21).

To our knowledge, there are few studies investigating peripheral blood immune cells and liver fat in large, population-based cohorts. In the present study, no relationships were observed between monocyte or T helper cell subsets with the presence of liver fat as assessed using an L/S attenuation ratio <1.0 by CT scan (29). These null results are consistent with some prior hepatology clinical studies (18, 19, 41), but not all (37). Although not meeting *a priori* significance thresholds, an association of higher memory CD4^+^ T cells was observed with >30% liver fat content (defined by liver attenuation <40 HU). This result is consistent with studies reporting higher circulating proportions of memory and lower proportions of naive CD4^+^ and CD8^+^ T cells in patients with MASH and higher CD4^+^ memory cells with features of metabolic syndrome in apparently healthy men (18, 42). Similarly, prior results from MESA identified a pattern of higher memory and lower naive CD4^+^ cells with type 2 diabetes and subclinical atherosclerosis (31, 32). Collectively, these results suggest that chronic T-cell stimulation from intrahepatic antigens, or those related to MAFLD comorbidities, promotes inflammation during hepatic steatosis and the progression to steatohepatitis, consistent with a role of chronic adaptive immune activation in MAFLD. The associations observed in the present study, however, were weak, the possibility of a type I error cannot be excluded, and results should be interpreted cautiously. Studies evaluating the relationships of activated, effector, and memory adaptive immune cell subsets across different stages of MAFLD remain important.

In exploratory analyses, higher CD8^+^CD57^+^ T cells were associated with both the presence of liver fat and with a liver fat content >30% when using a more lenient significance threshold criteria that did not correct for multiple comparisons. These results were supported by associations of the highly correlated phenotypes CD8^+^CD28^−^ and CD8^+^CD28^−^CD57^+^ cells with >30% liver fat content in models adjusted for MAFLD risk factors. CD57 expression on CD8^+^ T cells occurs in response to repeated antigen stimulation, often with the concomitant loss of CD28 expression, and is a marker of differentiation and senescence (43). CD8^+^CD57^+^ cells are implicated in autoimmunity, alcoholism, and age-related diseases including cancer (43), and their involvement in MAFLD was indicated in two prior seminal studies (44, 45).

In previous studies, elevated CD8^+^ T cells were reported in the liver and peripheral blood of patients with MAFLD (18, 46) and depletion of CD8^+^ T cells reduced progression of steatohepatitis in mice fed an obesogenic diet (14). In obese mice administered bromodichloromethane to induce oxidative stress, CD57 mRNA expression was increased 80-fold in the liver and CD8^+^ T cells were identified as the major subset expressing CD57. CD57 expression on CD8^+^ cells was increased in a CYP2E1- and leptin-dependent manner and accompanied by concomitant inflammatory cytokine secretion, hepatic apoptosis, and fibrogenesis during the progression of steatohepatitis (44). In a recent study by Sim at al., higher levels of CD8^+^CD28^−^CD57^+^ and CD4^+^CD28^−^CD57^+^ cells were observed in the livers of patients with type 2 diabetes and MASH or liver cirrhosis, compared with healthy controls, and correlated with the severity of liver fibrosis (45). Our results extend these prior findings to a large, population-based cohort with subclinical disease and suggest higher frequencies of CD8^+^CD57^+^ cells may reflect the early stages of MAFLD. The associations of CD8^+^CD57^+^ cells observed in this study, however, were from exploratory analyses and may reflect a type I error. These exploratory analyses did not account for multiple comparisons, and replication in other cohorts is required.

Limitations of the study include the case–cohort design which sampled for incident CVD cases and not incident MAFLD. The cross-sectional analyses cannot establish directionality, and we may have been underpowered to detect small or moderate associations with CT-assessed liver fat parameters. It is expected that a majority of those with liver fat in this study had subclinical disease, which may not be as strongly associated with peripheral immune cells compared with more advanced clinical stages of disease or with immune cells resident in hepatic tissue. Results from MESA participants, with an expected overall low severity of MAFLD, were not verified in clinical samples, which will be important to evaluate in future studies. MESA does not have biomarkers commonly used in the evaluation of liver diseases, such as liver enzymes or information on eating disorders, inborn impairment of metabolism, or hepatitis C status. Furthermore, current guidelines for MAFLD do not have CT criteria. Therefore, results from the current study must be interpreted in the context of CT-based liver fat assessment and cannot be generalized to current MAFLD guidelines (47, 48). We also cannot distinguish hepatic steatosis from steatohepatitis by CT imaging. Liver biopsy, however, is not practical in large epidemiological cohort studies.

Strengths of the study include the large panel of immune cells evaluated among a multiethnic, community-based cohort of asymptomatic participants. The use of CT imaging, which can be used to non-invasively assess liver fat in clinical and research settings, with standardized protocols and a central reading center is another strength.

In conclusion, results from this study suggest that the frequencies of monocyte and T helper cell subsets in peripheral blood are not strongly related with liver fat as assessed by CT imaging and may have limited utility as biomarkers of early-stage fatty liver. Memory CD4^+^ T cells may reflect liver fat severity, and CD8^+^CD57^+^ cells may be associated with the presence and severity of liver fat, but these findings require confirmation in other cohorts.

## Data availability statement

The data that support the findings of this study are available from the Collaborative Health Studies Coordinating Center (CHSCC) upon approval of a paper proposal using this data by the Multi-Ethnic Study of Atherosclerosis (MESA). The instructions are at https://www.mesa-nhlbi.org/. 

## Ethics statement

The University of Washington IRB Committee J reviewed the MESA cohort study and approved it as FWA #00006878. The studies were conducted in accordance with the local legislation and institutional requirements. The participants provided their written informed consent to participate in this study.

## Author contributions

RN performed statistical analysis and drafted and revised the manuscript. JD curated data, performed statistical analysis, and edited the manuscript. MD conceived the methodology, generated data, and edited the manuscript. AS performed statistical analysis and reviewed the manuscript. CS curated data and edited the manuscript. PB curated data, performed statistical analysis, and edited the manuscript. IZ generated data and edited the manuscript. RT conceived the study and reviewed the manuscript. BP conceived the study and edited the manuscript. MB conceived the study, generated data, and reviewed the manuscript. NO conceived the study, drafted and revised the manuscript. All authors agree to be accountable for the content of the work. All authors contributed to the article and approved the submitted version.
