# Supplementary material for: Investigating peripheral blood monocyte and T-cell subsets as non-invasive biomarkers for asymptomatic hepatic steatosis: results from the Multi-Ethnic Study of Atherosclerosis
Source: Front Immunol. 2024 Mar 26;15:1243526. doi: 10.3389/fimmu.2024.1243526 (PMC11002077; doi:10.3389/fimmu.2024.1243526)
Supplement: Supplementary file 1 [file DataSheet_1.pdf]

Supplementary Material

Investigating peripheral blood monocyte and T cell subsets as non-invasive biomarkers for asymptomatic hepatic steatosis: results from the Multi-Ethnic Study of Atherosclerosis

Rhys W. Niedecker, Joseph A. Delaney, Margaret F. Doyle, Andrew D. Sparks, Colleen M. Sitlani, Petra Buzkova, Irfan Zeb, Russell P. Tracy, Bruce M. Psaty, Matthew J. Budoff, and Nels C. Olson*

*** Correspondence:** Nels C. Olson, PhD, MPH: nels.olson@med.uvm.edu

Supplemental Table 1. Characterization of the immune cell phenotypes included in the analysis

Cell Phenotype	Molecular Markers	Parent population	n	Mean (SD) (%)
Classical Monocytes	$CD14^{++}CD16^{-}$	$CD14^{+}$ monocytes	1557	75.1 (10.4)
Non-Classical Monocytes	$CD14^{+}CD16^{++}$	$CD14^{+}$ monocytes	1557	9.2 (7.6)
Intermediate monocytes	$CD14^{+}CD16^{+}$	$CD14^{+}$ monocytes	1557	15.8 (7.6)
γδ T	$CD3^{+}\gamma\delta TCR^{+}$	$CD3^{+}$ cells	1790	5.9 (5.8)
Natural killer (NK)	$CD3^{-}CD16^{+}CD56^{+}$	Lymphocytes	1790	6.2 (6.1)
Th1	$CD4^{+}IFN\text{-}\gamma^{+}$	$CD4^{+}$ T cells	1552	13.9 (7.9)
Th2	$CD4^{+}IL\text{-}4^{+}$	$CD4^{+}$ T cells	1552	2.7 (1.9)
Th17	$CD4^{+}IL\text{-}17A^{+}$	$CD4^{+}$ T cells	1552	2.6 (1.4)
T regulatory (Treg)	$CD4^{+}CD25^{+}CD127^{-}$	$CD4^{+}$ T cells	1764	4.3 (2.2)
Naive $CD4^{+}$	$CD4^{+}CD45RA^{+}$	$CD4^{+}$ T cells	1839	26.8 (12.7)
Memory $CD4^{+}$	$CD4^{+}CD45RO^{+}$	$CD4^{+}$ T cells	1839	56.4 (14.7)
Activated or Treg $CD4^{+}$	$CD4^{+}CD25^{+}$	$CD4^{+}$ T cells	1765	28.3 (11.3)
Activated or mature $CD4^{+}$	$CD4^{+}CD57^{+}$	$CD4^{+}$ T cells	1839	17.3 (12.8)
Differentiated / senescent $CD4^{+}$	$CD4^{+}CD28^{-}$	$CD4^{+}$ T cells	1839	10.7 (10.1)
Differentiated / senescent $CD4^{+}$	$CD4^{+}CD28^{-}CD57^{+}$	$CD4^{+}$ T cells	1844	7.9 (8.4)
TEMRA $CD4^{+}$	$CD4^{+}CD28^{-}CD57^{+}CD45RA^{+}$	$CD4^{+}$ T cells	1839	3.9 (4.9)
Tc1	$CD8^{+}IFN\text{-}\gamma^{+}$	$CD8^{+}$ T cells	1489	35.9 (17.2)
Tc2	$CD8^{+}IL\text{-}4^{+}$	$CD8^{+}$ T cells	1489	4.0 (4.6)
Tc17	$CD8^{+}IL\text{-}17A^{+}$	$CD8^{+}$ T cells	1489	3.3 (4.8)
Naive $CD8^{+}$	$CD8^{+}CD45RA^{+}$	$CD8^{+}$ T cells	1869	55.9 (15.3)
Memory $CD8^{+}$	$CD8^{+}CD45RO^{+}$	$CD8^{+}$ T cells	1869	23.7 (11.7)
Differentiated / senescent $CD8^{+}$	$CD8^{+}CD28^{-}$	$CD8^{+}$ T cells	1849	58.2 (15.8)
Differentiated / senescent $CD8^{+}$	$CD8^{+}CD57^{+}$	$CD8^{+}$ T cells	1849	57.7 (16.3)
Differentiated / senescent $CD8^{+}$	$CD8^{+}CD28^{-}CD57^{+}$	$CD8^{+}$ T cells	1874	46.7 (15.9)
TEMRA $CD8^{+}$	$CD8^{+}CD28^{-}CD57^{+}CD45RA^{+}$	$CD8^{+}$ T cells	1849	34.8 (14.4)

TEMRA, T effector memory RA^{+}; Tc, Cytotoxic T cell; TCR, T cell receptor; Th, T helper

Supplemental Table 2. Sensitivity analyses of immune cell subsets with liver-to-spleen ratios <1 HU and liver attenuation values <40 HU with adjustment for cytomegalovirus (CMV) antibody titers

Cell subset	Liver-to-Spleen Ratio <1.0 OR (95% CI)	P-value	Liver Attenuation <40 HU OR (95% CI)	P-value
Classical Monocytes	0.83 (0.68, 1.02)	0.08	0.97 (0.75, 1.26)	0.81
Non-Classical Monocytes	1.11 (0.92, 1.34)	0.26	1.04 (0.81, 1.32)	0.78
Naive $CD4^+$	1.00 (0.84, 1.20)	0.96	0.83 (0.67, 1.04)	0.11
Memory $CD4^+$	1.06 (0.87, 1.30)	0.57	1.27 (1.00, 1.61)	0.05
Th1	0.99 (0.81, 1.22)	0.96	0.98 (0.76, 1.25)	0.85
Th17	0.88 (0.76, 1.08)	0.24	0.99 (0.78, 1.25)	0.90
γδ	0.84 (0.70, 1.01)	0.07	1.18 (0.96, 1.45)	0.12
Natural Killer	0.91 (0.74, 1.14)	0.42	0.98 (0.77, 1.25)	0.87
Intermediate Monocyte	1.17 (0.92, 1.49)	0.19	1.01 (0.74, 1.37)	0.95
Th2	0.89 (0.72, 1.11)	0.30	1.07 (0.86, 1.33)	0.54
Treg	0.82 (0.64, 1.05)	0.12	1.13 (0.84, 1.53)	0.42
$CD4^+CD25^+$	1.04 (0.84, 1.28)	0.72	1.05 (0.80, 1.38)	0.73
$CD4^+CD28^-$	0.84 (0.66, 1.07)	0.16	1.11 (0.84, 1.47)	0.47
$CD4^+CD57^+$	0.91 (0.74, 1.12)	0.39	1.03 (0.79, 1.35)	0.82
$CD4^+CD28^-CD57^+$	0.88 (0.71, 1.10)	0.25	1.07 (0.83, 1.38)	0.60
$CD4^+$ TEMRA	0.89 (0.71, 1.11)	0.30	1.08 (0.83, 1.41)	0.56
Tc1	0.98 (0.79, 1.22)	0.86	0.99 (0.72, 1.37)	0.96
Tc2	0.80 (0.63, 1.03)	0.08	0.95 (0.68, 1.33)	0.76
Tc17	0.95 (0.76, 1.19)	0.67	1.00 (0.73, 1.36)	0.99
Naive $CD8^+$	1.04 (0.88, 1.23)	0.68	1.10 (0.85, 1.41)	0.47
Memory $CD8^+$	0.90 (0.76, 1.07)	0.23	0.93 (0.73, 1.18)	0.53
$CD8^+CD28^-$	1.05 (0.88, 1.25)	0.59	1.45 (1.11, 1.90)	0.006
$CD8^+CD57^+$	1.22 (1.03, 1.46)	0.03	1.40 (1.11, 1.77)	0.005
$CD8^+CD28^-CD57^+$	1.16 (0.97, 1.39)	0.11	1.47 (1.14, 1.90)	0.003
$CD8^+$ TEMRA	1.10 (0.91, 1.32)	0.32	1.27 (1.00, 1.63)	0.05

Analyses are by logistic regression models and include sampling weights to account for the case-cohort study design. Cells were analyzed in separate models per 1-SD higher value. A liver-to-spleen ratio Hounsfield units (HU) <1.0 was used to diagnose presence of liver fat. A liver attenuation <40 HU was assessed as a cutoff of >30% liver fat content.

Models adjusted for age, sex, race/ethnicity, education, analytical batch, waist circumference, alcohol use, diabetes status, statin use, hypertensive medication use, systolic blood pressure, HDL, LDL, triglycerides, and CMV antibody titer. Results in highlighted cells are referenced in the main manuscript text and were near or below the significance threshold as defined for the exploratory analyses.

Tc, Cytotoxic T cell; TEMRA, T effector memory RA^+.
